# Flavan-3-ol Compounds from Wine Wastes with *in Vitro* and *in Vivo* Antioxidant Activity

**DOI:** 10.3390/nu2101048

**Published:** 2010-10-11

**Authors:** Gustavo Scola, Danusa Conte, Patrícia Wilmsen Dalla-Santa Spada, Caroline Dani, Regina Vanderlinde, Claudia Funchal, Mirian Salvador

**Affiliations:** 1 Instituto de Biotecnologia, Universidade de Caxias do Sul, 95070560 Caxias do Sul, RS, Brazil; Email: gustavo.scola@gmail.com (G.S.); danuconte@hotmail.com (D.C.); pspada@ucs.br (P.W.D.-S.S.); rvanderl@ucs.br (R.V.); 2 Curso de Biomedicina, Faculdade da Serra Gaúcha FSG, 95020472 Caxias do Sul, RS, Brazil; 3 Centro Universitário Metodista IPA, 90420060 Porto Alegre, RS, Brazil; Email: carolinedani@yahoo.com.br (C.D.); csfunchal@yahoo.com.br (C.F.)

**Keywords:** aqueous wine waste extracts, *V. vinifera*, *V. labrusca*, phenolic content, antioxidant

## Abstract

It has been suggested that the dietary intake of antioxidant supplements could be a useful strategy to reduce the incidence of diseases associated with oxidative stress. The aim of present work is to study the possibility to obtain compounds with antioxidant activity from wine wastes using water as solvent. Results have shown that it is possible to obtain flavan-3-ol compounds from wine wastes both from *V. vinifera* (cv. Cabernet Sauvignon and Merlot) and *V. labrusca* (cv. Bordo and Isabella) species. The main phenolic compounds found in the extracts were catechin and epicatechin, followed by procyanidin B3, procyanidin B1, procyanidin B2, gallic acid, epigallocatechin, and procyanidin B4. All flavan-3-ol extracts showed significant *in vitro* and *in vivo* activities. It was found that the extracts were able to prevent lipid and protein oxidative damage in the cerebral cortex, cerebellum and hippocampus tissues of rats. Although further studies are necessary, these flavan-3-ol extracts show potential to be used to reduce the incidence of degenerative diseases associated with oxidative stress.

## 1. Introduction

The role of dietary supplements in the prevention of some diseases has received widespread attention [1]. It has been suggested that the dietary intake of antioxidant supplements could be a useful strategy to reduce the incidence of diseases associated with oxidative stress, such as cancer, atherosclerosis and neurodegenerative diseases [2]. Vegetables, fruits and their seeds are rich sources of antioxidant compounds, such as vitamins, beta-carotene and polyphenols. Among the fruits, grapes have high polyphenol content, and 60–70% of these compounds are found in grape seeds. Grape seed supplements, obtained from *Vitis vinifera* species using organic solvents, have been reported to have a broad spectrum of pharmacological effects, such as antioxidative, anti-inflammatory and antimicrobial activities, as well as cardioprotective, hepatoprotective and neuroprotective effects [3].

Among the polyphenols found in grapes, flavonoids are one of the most abundant groups [4], including colorless flavan-3-ol compounds such as catechin, epicatechin and their polymers [5]. It has been shown that flavan-3-ols possess many biological effects including the scavenging of free radicals, chelation of transition metals, as well as the modulation of some antioxidant enzymes [6]. 

Some of the polyphenols present in grapes are extracted into wine, but most remain in the vinification wastes (pomace, stems and seeds), which account for about 13% of the processed grape weight [7]. Every year, the worldwide wine production (around 260 million hL) generates about 19.5 million ton of waste, which usually end up being used as fertilizer or simply being discarded [8]. *Vitis vinifera* (cv. Cabernet Sauvignon and Merlot) and *Vitis labrusca* (cv. Bordo and Isabella) are the main varieties used to produce wine and grape juices [9]. There are many works about the biological effects of grape seed extracts obtained from *V. vinifera* [10]. However, the possibility to use *V. labrusca* varieties as a source of polyphenols is not well established.

Different methods have been developed to measure the antioxidant activity of natural compounds. This evaluation can be performed by *in vitro* or *in vivo* assays, and it is suggested to conduct both of them to get more reliable results. Among the *in vitro* assays, the ability of one compound to donate electrons to the stable radical 2,2-diphenyl-1-picrylhydrazyl (DPPH**˙**) is one of the most used and reproducible assays [11]. 

Assays using mammalian living cells have also proven to be very useful in identifying antioxidant activity [12]. Brain cells are highly vulnerable to oxidative damage due to their high consumption of oxygen, the presence of large amounts of easily oxidizable polyunsaturated fatty acids, and an abundance of redox-active transition metal ions [13]. Lipid peroxidation in brain tissues is associated with a progressive loss of membrane permeability and cellular damage [13], leading to an increased susceptibility to various diseases, such as Parkinson’s and Alzheimer’s diseases [14]. Oxidative damage to lipids and proteins were evaluated in these tissues, as well as the enzymatic (catalase activity) and non-enzymatic (protein sulfhydryl assay) defenses found in mammalian cells [15].

The eukaryotic yeast *Saccharomyces cerevisiae* has been used to carry out *in vivo* assays, showing rapid and reproducible results. This yeast has been extensively studied both genetically and biochemically, and it is used for determining antioxidant activities [16,17]. 

The purpose of this study was to investigate the possibility to obtain flavan-3-ol compounds from winery waste seeds of *V. vinifera* (cv. Cabernet Sauvignon and Merlot) and *V. labrusca* (cv. Bordo and Isabella) using water as a solvent and also to evaluate their antioxidant activity using *in vitro* and *in vivo* assays.

## 2. Experimental Section

### 2.1. Chemicals

Procyanidin B3, (+)-catechin, (−)-epicatechin, (−)-epigallocatechin, gallic acid, 2,2-diphenyl-1-picrylhydrazyl (DPPH**˙**), 2,4-dinitrophenylhydrazine (DNPH), 5,5’-dithiobis(2-nitrobenzoic acid) (DTNB), thiobarbituric acid (TBA) were purchased from Sigma-Aldrich (St. Louis, MO). All other reagents were of analytical grade.

### 2.2. Wine waste extracts

Seeds from winery wastes of *V. vinifera* (cv. Cabernet Sauvignon and Merlot) and *V. labrusca* (cv. Bordo and Isabella) were removed from the vinification tanks five days after the beginning of fermentation in January 2006. All varieties were cultivated in the northeast region of the Serra Gaucha, Rio Grande do Sul, Brazil. Voucher specimens were identified by the herbarium of the University of Caxias do Sul, Rio Grande do Sul, Brazil (*V. vinifera* HUCS32455-32456 and *V. labrusca* HUCS31065-31066). Seeds were manually separated from the rest of the winery wastes, dried in air oven at 37 °C and stored at 25 °C sheltered from light. Extracts were obtained using 5 g of seeds/100 mL of distilled water, under reflux (100 °C), for 30 minutes. After cooling to 25 °C, extracts were filtered in Millipore equipment (pore size, 0.45 µm; catalog number SFGS 047LS, Millipore Corp., São Paulo, Brazil). The extracts were freeze-dried (Edward freeze dryer) at 60 °C, 10^−1^ bar, and were stored at −20 °C. All grape seed extracts were solubilized in distilled water immediately before use.

### 2.3. Phenolic content of the wine waste extracts

Total phenolic content of the wine waste extracts were measured using Singleton and Rossi’s (1965) modification of the Folin–Ciocalteu colorimetric method [18]. Two hundred microliters of the extracts were assayed with 1000 μL of Folin–Ciocalteu reagent and 800 μL of sodium carbonate (7.5%, w/v). The mixture was vortexed and diluted (1:10) with distilled water. After 30 minutes, the absorbance was measured at 765 nm, and the total phenolic content was expressed as mg/L catechin equivalent (CTE). Major polyphenols were assessed through chromatographic analyses carried out as described by Lamuela-Raventós and Waterhouse (1994) [19] using a HP 1100 (Palo Alto, CA) diode array UV-visible detector coupled to an HP Chem Station. A Zorbax SB C18 (250 × 4 mm), 5 μm particle size, was used for the stationary phase with a flow of 0.5 mL/min. Twenty-five microliters of extracts were injected into the HPLC system after filtration through a 0.45 μm Millipore membrane. The solvents used for the separation were as follows: solvent A (50 mM dihydrogen ammonium phosphate adjusted to pH 2.6 with orthophosphoric acid), solvent B (20% of solvent A with 80% acetonitrile) and solvent C (0.2 M orthophosphoric acid adjusted with ammonia to pH 1.5). The gradient conditions were: solvent A 100% (0–5 min), solvents A 96% and B 4% (5–15 min), solvents A 92% and B 8% (15–25 min), solvents B 8% and C 92% (25–45 min), solvents B 30% and C 70% (45–50 min), solvents B 40% and C 60% (50–55 min), solvents B 80% and C 20% (55–60 min) and solvent A 100% (60–65 min). Chromatograms were monitored at 204 nm, and identification was based on retention times relative to authentic standards ((+)-catechin, (−)-epicatechin, (−)-epigallocatechin, procyanidin B1, B2, B3, and B4, and gallic acid). Quantification was performed using the standards by establishing calibration curves for each identified compound. Results are shown in mg/L.

### 2.4. *In vitro* antioxidant activity

*In vitro* antioxidant activity of the different wine waste extracts was measured by 2,2-diphenyl-1-picrylhydrazyl radical (DPPH**˙**) scavenging activity [11], catalase-like assay [15], and in brain tissue of rats. For the DPPH**˙** assay, the extracts were added to Tris-HCl buffer (100 mM, pH 7.0) containing 250 μM DPPH**˙** dissolved in ethanol. The tubes were kept in the dark for 20 min and absorbance was measured at 517 nm (UV-1700 spectrophotometer, Shimadzu, Kyoto, Japan). Results were calculated as IC_50_ (amount of extract necessary to scavenge 50% of DPPH**˙** radical). Catechin was used as a control.

To assess the antioxidant activity in brain tissue, ten-day-old Wistar rats were obtained from the breeding colony of the Centro Universitário Metodista. They were maintained at approximately 25 °C, on a 12-h light/12-h dark cycle. All efforts were made to minimize animal suffering and to use only the number of animals necessary to produce reliable scientific data. The experiments were performed in accordance with “Guide for the Care and Use of Laboratory Animals, DHEW, publication no. (NIH) 85-23, 1985” and approved by the local ethical committee at Universidade de Caxias do Sul. Assays were performed as described by Leipnitz *et al.* [20]. Briefly, animals were killed by decapitation without anesthesia, and the brain was rapidly excised on a Petri dish placed on ice. The cerebral cortex, cerebellum and hippocampus were dissected, weighed and kept chilled until homogenization, which was performed using a ground-glass-type Potter-Elvehjem homogenizer in 1.5% KCl. The homogenates were centrifuged (800 g) for 10 min at 4 °C, the pellets were discarded and the supernatants were used immediately. Aliquots were treated with the wine waste extracts (1.5%, v/v) for 30 min and then 5 mM hydrogen peroxide (H_2_O_2_) was added to the mixture. Samples were incubated for 1 h at 37 °C under constant agitation. All experiments were conducted in accordance with the Guiding Principles of the Use of Animals in Toxicology, adopted by the Society of Toxicology in July 1989. 

Oxidative markers analyses included the quantification of lipid and protein damages and the activity of the antioxidant enzyme catalase. Protein sulfhydryl content was assessed as a non-enzymatic cellular defense. Lipid damages were monitored by the formation of thiobarbituric acid reactive species (TBARS) during an acid-heating reaction, which has been widely adopted as a sensitive method for measuring lipid peroxidation. First, 1000 μL of 5% trichloroacetic acid were added to 250 μL of supernatants and centrifuged at 7000 g for 10 min. Then, 1000 μL of sulfuric acid (3 M) were mixed with 1000 μL of thiobarbituric acid solution. The reaction mixture was incubated in a boiling water bath for 15 min, and cooled to room temperature. Then, 3500 μL of n-butanol were added and centrifuged at 7000 g for 5 min. The absorbance was read at 532 nm [21]. Results are expressed as nmol of TBARS/mg of protein. Oxidative damage in proteins was measured by determining the carbonyl grouping based on the reaction with dinitrophenylhydrazine (DNPH). Two hundred microliters of DNPH (10 mM) or 200 μL of HCL (2 M) for control were added to 50 μL of supernatants. The reaction mixture was incubated in the dark for 30 minutes, with vortex every 10 minutes; after that, 250 μL of 20% trichloroacetic acid were added and centrifuged at 4000 g for 8 minutes. The supernatant was discarded and the pellet was washed 3 times with ethanol-ethyl acetate (1:1) to remove free reagent. Samples were centrifuged and pellets were redissolved in 600 μL of guanidine solution (6 M) at 37 °C for 15 minutes. Absorbance was read at 365 nm [22], and results expressed as nmol of DNPH/mg of protein. Catalase activity was determined by the hydrogen peroxide decomposition rate. Briefly, 20 μL of the wine waste extracts were added to 2910 μL phosphate buffer (pH 7.4) plus 70 μL of H_2_O_2_ (3 mM freshly diluted) and read on a spectrophotometer at 240 nm and values were expressed as µmol of H_2_O_2_/per minute per mg of protein [23]. Determination of protein sulfhydryl content was based on the reaction with 5,5’-dithiobis(2-nitrobenzoic acid) (DTNB) whose absorbance was measured spectrophotometrically at 412 nm. Briefly, 0.1 mM DTNB was added to 120 μL of supernatants. This was followed by 30 min incubation at room temperature in a dark room. The sulfhydryl content is inversely correlated with oxidative damage to proteins. Results are expressed as µmol of DTNB/mg of protein [24]. Protein concentration was determined by the Bradford method [25] using bovine serum albumin as standard. 

### 2.5. *In vivo* antioxidant activity

*In vivo* antioxidant activity was carried out using eukaryotic cells of the yeast *Saccharomyces cerevisiae* XV185-14C (*MATα*, *ade 2-1*, *arg 4-17*, *his 1-7*, *lys 1-1*, *trp 1-1*, *trp 5-48*, *hom 3-10*) provided by Dr. R.C. Von Borstel (Genetics Department, University of Alberta, Edmonton, AB, Canada) treated with the highest noncytotoxic concentration, 2.5% (v/v), as well as 0.5% (v/v) and 1.5% (v/v) of each extract plus H_2_O_2_ (4 mM). The tubes were incubated for 2 h at 28 °C. The samples were diluted in a sodium chloride solution 0.9% (p/v), seeded into a complete culture medium (10 g/L of yeast extract, 20 g/L of peptone, 20 g/L of dextrose and 20 g/L of agar-agar) and incubated for 48 h at 28 °C. After incubation, the colonies were counted, defining the total number of colonies observed on the control plate (untreated cells) as a 100% survival rate. The antioxidant activity of the extracts was evaluated by the ability of the extracts to avoid/minimize the oxidative lethal damages induced by H_2_O_2_, as already described [16,17].

### 2.6. Statistical analysis

All measurements were performed at least in triplicate, and values were averaged and reported along with the standard deviation. Data were subjected to analysis of variance (ANOVA), Tukey test, and Pearson correlation using a SPSS 12.0 software package (SPSS Inc., Chicago, IL). 

## 3. Results and Discussion

The total phenolic content of the aqueous wine waste extracts studied in this work varied from 353.20 ± 4.60 mg/L to 751.38 ± 5.30 mg/L for the Isabella and Merlot varieties, respectively. Cabernet Sauvignon, Merlot and Bordo extracts did not show any significant differences in total phenolic content. The main phenolic compounds in all wine waste extracts were catechin and epicatechin, followed by procyanidin B3, procyanidin B1, procyanidin B2, gallic acid, epigallocatechin, and procyanidin B4 (Table 1). This work shows that it is possible to obtain flavan-3-ol compounds from wine waste seeds using water as a solvent. This aspect is important to avoid possible organic solvent residues in the final product. It is important to mention that the aqueous extracts of *V. vinifera* studied herein present similar phenolic composition to that observed in seed extracts from Cabernet Sauvignon and Merlot varieties prepared in ethanol [26], methanol [27] and ethyl acetate/acetone [28]. Besides, we have shown here that it is possible to use *V. labrusca*, mainly Bordo variety, as source of flavan-3-ol compounds.

**Table 1:** Total polyphenol content (mg/L equivalent of catechin) and major compounds (mg/L) in the wine waste extracts.

		Major compounds							
GSE	TPC	CT	ECT	EGC	B1	B2	B3	B4	GA
Cabernet Sauvignon	715.59 ± 5.87 ^a^	106.73 ± 0.34 ^a^	71.53 ± 0.33 ^a^	8.14 ± 1.29 ^a^	26.54 ± 1.86 ^ab^	15.23 ± 0.08 ^a^	29.53 ± 2.70 ^a^	2.89 ± 0.02 ^a^	11.87 ± 0.17 ^a^
Merlot	751.38 ± 5.30 ^a^	109.57 ± 0.20 ^a^	111.08 ± 0.05 ^b^	7.49 ± 0.97 ^a^	27.80 ± 0.82 ^b^	13.73 ± 0.17 ^a^	47.16 ± 0.45 ^b^	2.87 ± 0.19 ^a^	16.42 ± 1.15 ^b^
Bordo	744.89 ± 3.13 ^a^	169.26 ± 0.92 ^b^	168.86 ± 2.82 ^c^	8.96 ± 0.05 ª	22.42 ± 0.51 ª	19.75 ± 0.17 ª	17.45 ± 0.01 ^c^	1.85 ± 0.12 ^ab^	12.98 ± 0.54 ª
Isabella	353.20 ± 4.60 ^b^	135.36 ± 0.99 ^c^	112.40 ± 0.32 ^b^	5.64 ± 0.02 ^b^	8.86 ± 0.03 ^c^	3.17 ± 3.64 ^b^	9.72 ± 0.01 ^d^	1.72 ± 0.06 ^b^	6.88 ± 0.04 ^c^

Results represent average values ± S.D. ^a^ Different letters indicate significant differences using analysis of variance (ANOVA) and Tukey post-hoc test (p ≤ 0.05). TPC: total phenolic content; CT: catechin; ECT: epicatechin; EGC: epigallocatechin; B1: procyanidin B1; B2: procyanidin B2; B3: procyanidin B3; B4: procyanidin B4; GA: gallic acid.

**Figure 1:**
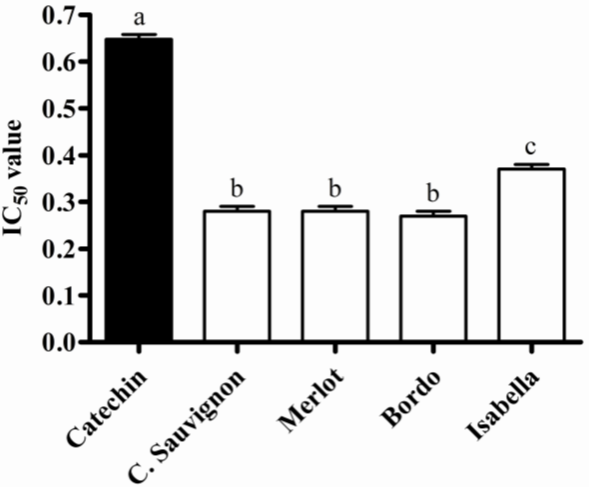
*In vitro* antioxidant activity of wine waste extracts. IC_50_ is the amount (%) of extracts needed to scavenge 50% of DPPH**˙**. Catechin was used as control. The results represent mean ± S.D. of three independent experiments. Different letters are statistically different by analysis of variance (ANOVA) and Tukey’s post-hoc test (p ≤ 0.05).

DPPH**˙** assay [11] was used to assess *in vitro* antioxidant activity of the wine waste extracts. All of them showed higher antioxidant activity than the standard catechin. The extracts with high levels of polyphenols content (Bordo, Cabernet Sauvignon and Merlot) also showed higher antioxidant activity (Figure 1). In fact, a strong correlation was found (r^2^ = 0.950, p ≤ 0.01) between the total phenolic content and the DPPH**˙** assay, suggesting that these compounds are responsible, at least in part, for the antioxidant activity observed. 

Brain cells are continuously threatened by the damage caused by reactive oxygen species produced during normal oxygen metabolism or induced by exogenous sources [29]. The cerebral cortex, cerebellum and hippocampus are known to be the major integrative parts affected in various neurodegenerative disorders [30], such as amyotrophic lateral sclerosis, Alzheimer’s and Parkinson’s diseases [31,32,33]. The cerebral cortex and the hippocampus are regions associated with cognition and feedback stress control, while the cerebellum is uncharged of motor function [34]. Therefore, the biological effects of wine waste extracts in brain tissues were also studied. Results show that treatments with hydrogen peroxide induced an increase in lipid (TBARS) and protein (carbonyl) damages and in catalase activity, along with a decrease in protein sulfhydryl content in all tissues analyzed. Pre-treatment with the wine waste extracts prevented lipid and protein damages (Table 2), and the increase in catalase activity induced by H_2_O_2_, as well as protected the sulfhydryl content from oxidation in the cerebral cortex, cerebellum and hippocampus of rats (Table 3).

**Table 2:** Lipid and protein damage in the cerebral cortex, hippocampus and cerebellum of rats treated with the wine waste extracts plus hydrogen peroxide.

Treatments	Lipid damage (nmol/mg protein)				Protein damage (nmol/mg protein)		
	Cerebral Cortex	Cerebellum	Hippocampus		Cerebral Cortex	Cerebellum	Hippocampus
Control	0.89 ± 0.02 ª	1.16 ± 0.17 ª	1.96 ± 0.11 ª		29.06 ± 3.16 ª	3.45 ± 0.54 ª	1.06 ± 0.30 ª
H_2_O_2_	1.93 ± 0.03 ^b^	1.84 ± 0.09 ^d^	5.03 ± 0.09 ^b^		64.37 ± 1.26 ^b^	7.86 ± 0.27 ^b^	3.06 ± 0.30 ^b^
Cabernet Sauvignon + H_2_O_2_	0.27 ± 0.03 ^c^	0.70 ± 0.08 ^c^	1.89 ± 0.41 ^ac^		10.73 ± 1.27 ^c^	5.18 ± 0.81 ª	0.85 ± 0.20 ª
Merlot + H_2_O_2_	0.22 ± 0.03 ^c^	0.76 ± 0.10 ^bc^	1.36 ± 0.47 ^ac^		11.18 ± 0.63 ^c^	3.45 ± 0.54 ª	1.07 ± 0.10 ª
Bordo + H_2_O_2_	0.38 ± 0.05 ^d^	0.99 ± 0.06 ^ab^	1.37 ± 0.25 ^c^		14.75 ± 4.42 ^c^	4.41 ± 0.81 ª	0.71 ± 0.10 ª
Isabella + H_2_O_2_	0.46 ± 0.03 ^d^	1.04 ± 0.13 ^a^	2.12 ± 0.13 ^a^		14.75 ± 0.63 ^c^	4.60 ± 0.54 ª	0.57 ± 0.10 ª

Tissues were incubated for 30 min in the presence of the different extracts (1.5%) and 1 h in the presence of 5 mM H_2_O_2_. Data are mean ± S.D. Different letters indicate a significant difference according to analysis of variance and Tukey’s post-hoc test (p ≤ 0.05) for each tissue evaluated.

**Table 3:** Enzymatic and non-enzymatic defenses in cerebral cortex, hippocampus and cerebellum of rats treated with the wine waste extracts plus hydrogen peroxide.

Treatments	Catalase (µmol H_2_O_2_/mg protein/min)				Protein sulfhydryl content (µmol/mg protein)		
	Cerebral Cortex	Cerebellum	Hippocampus		Cerebral Cortex	Cerebellum	Hippocampus
Control	0.06 ± 0,01 ª	0.18 ± 0,01 ª	0.33 ± 0,01 ª		27.54 ± 1.13 ^a^	21.39 ± 1.30 ^a^	48.91 ± 1.70 ^a^
H_2_O_2_	0.09 ± 0.01 ^b^	0.26 ± 0.01 ^b^	0.62 ± 0.01 ^b^		17.56 ± 1.69 ^b^	13.95 ± 1.32 ^b^	26.33 ± 1.77 ^b^
Cabernet Sauvignon + H_2_O_2_	0.06 ± 0.01 ^a^	0.20 ± 0.01 ^c^	0.06 ± 0.01 ^ac^		27.94 ± 1.69 ^a^	33.48 ± 0.01 ^c^	31.35 ± 1.70 ^c^
Merlot + H_2_O_2_	0.02 ± 0.01 ^d^	0.08 ± 0.01 ^d^	0.02 ± 0.01 ^cd^		25.94 ± 1.13 ^a^	31.62 ± 2.63 ^c^	36.62 ± 1.42 ^c^
Bordo + H_2_O_2_	0.05 ± 0.01 ^ac^	0.07 ± 0.01 ^e^	0.05 ± 0.01 ^ac^		23.15 ± 2.82 ^ab^	37.20 ± 2.60 ^c^	55.18 ± 3.55 ^c^
Isabella + H_2_O_2_	0.03 ± 0.01 ^cd^	0.10 ± 0.01 ^f^	0.03 ± 0.01 ^cd^		29.93 ± 1.13 ^a^	38.13 ± 1.32 ^c^	37.44 ± 0.25 ^c^

Tissues were incubated for 30 min in the presence of the different extracts (1.5%) and 1 h in the presence of 5 mM H_2_O_2_. Data are mean ± S.D. Different letters indicate a significant difference according to analysis of variance and Tukey’s post-hoc test (p ≤ 0.05) for each tissue evaluated.

The TBARS assay is one of the oldest and most frequently used tests for measuring the peroxidation of fatty acids and membranes. The main product formed during lipid peroxidation is malondialdehyde, a powerful genotoxic and carcinogenic compound [14]. Proteins are also target of oxidative modification by reactive oxygen species. These reactions often lead to the modification of certain amino acid residues forming carbonyl derivates, which is linked to losses in physiological functions under pathological processes or during aging [35]. 

Pearson’s correlations between the oxidative stress markers in the cerebral cortex, cerebellum and hippocampus tissues and the phenolic content levels of the different extracts are shown in Table 4. In a general way, it is possible to observe that polyphenols present negative correlations with lipid and protein oxidative damages, suggesting that these compounds are able to prevent the damages induced by H_2_O_2_.

**Table 4:** Pearson correlations and their statistical significance among the wine waste extract constituents and oxidative parameters evaluated.

	TPC	CT	ECT	EGC	B1	B2	B3	B4	GA
Cerebral cortex lipid damage	−0.752 ^**^	−0.686 ^*^	−0.616 ^*^	−0.755 ^**^	−0.745 ^**^	−0.586 ^*^	−0.719 ^**^	−0.833 ^**^	−0.759 ^**^
Cerebellum lipid damage	−0.794 ^**^	−0.650 ^*^	−0.587 ^*^	−0.765 ^**^	−0.801 ^**^	−0.682 ^*^	−0.744 ^**^	−0.840 ^**^	−0.780 ^**^
Hippocampus lipid damage	−0.718 ^**^	−0.608 ^*^	−0.634 ^*^	−0.665 ^*^	−0.706 ^*^	−0.617 ^*^	−0.725 ^**^	−0.696 ^**^	−0.769 ^**^
Cerebral cortex protein damage	−0.781 ^**^	−0.771 ^**^	−0.714 ^**^	−0.803 ^**^	−0.746 ^**^	−0.658 ^*^	−0.654 ^*^	−0.804 ^**^	−0.769 ^**^
Hippocampus protein damage	−0.580 ^*^	−0.694 ^*^	−0.640 ^*^	−0.656 ^*^	n.f.	n.f.	n.f.	−0.586 ^*^	n.f.

TPC: total phenolic content; CT: catechin; ECT: epicatechin; EGC: epigallocatechin; B1: procyanidin B1; B2: procyanidin B2; B3: procyanidin B3; B4: procyanidin B4; GA: gallic acid; n.f.: not found. * Significant Pearson correlation for p ≤ 0.05 and ** for p ≤ 0.01.

**Figure 2:**
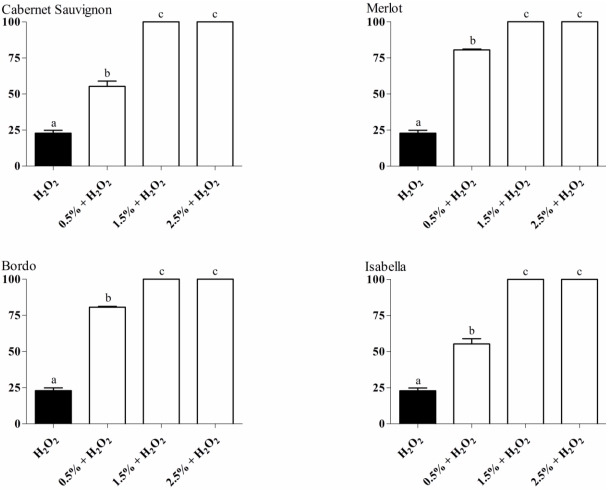
Survival of *S. cerevisiae* cells treated with wine waste extracts plus hydrogen peroxide. Data are mean ± S.D. Different letters indicate significant differences using analysis of variance (ANOVA) and Tukey’s post-hoc test (p ≤ 0.05).

To assess the *in vivo* antioxidant activity of wine waste extracts, eukaryotic cells of *S. cerevisiae* treated with noncytotoxic concentrations of the extracts plus H_2_O_2_ were used. Results (Figure 2) show that all extracts were able to protect the yeast cells against the damage induced by H_2_O_2_. At higher concentrations (1.5 and 2.5%) of the extracts, the wine waste extracts completely prevented (100% survival) the cytotoxic effects of H_2_O_2_. The following positive correlations between *in vivo* antioxidant activity and total phenolic content for each extract were found: Bordo (r^2^ = 0.877, p ≤ 0.05), Isabella (r^2^ = 0.847, p ≤ 0.05), Cabernet Sauvignon (r^2^ = 0.867, p ≤ 0.05) and Merlot (r^2^ = 0.935, p ≤ 0.05), suggesting the role of these compounds in the *in vivo* antioxidant activity observed in this work.

The antioxidant mechanisms of the phenolic compounds are complex and are still being studied. In general, they can prevent the formation of reactive species by chelating trace elements involved in free radical production, scavenge reactive species, and upregulate or protect antioxidant defenses [14]. 

Apart from known vitamins and minerals, phenolic compounds may be one of the most widely marketed groups of dietary supplements. This class of plant metabolites shows antibacterial effects [36], an ability to reduce blood pressure [37] and antioxidant, anti-inflammatory, antimutagenic and/or anticarcinogenic effects, at least in *in vitro* systems [38,39,40]. 

Supplementation of bioavailable and safe natural products, as polyphenols, is important to complement diets poor in antioxidants that we consume daily. Although further studies are necessary, the flavan-3-ol compounds studied herein show potential to be used as antioxidants. Studies about the safety of these extracts are being conducted, but it is already known that procyanidin extracts from grape seeds, assessed in compliance with the U.S. Environmental Protection Agency’s Health Effects Test Guidelines and the Toxic Substances Control Act, have shown to be safe for human intake [41].

## 4. Conclusions

The data presented herein has shown that it is possible to obtain flavan-3-ol compounds from wine wastes using water as a solvent. As we hypothesized, both *V. vinifera* and *V. labrusca* species can be used to obtain extracts with important *in vitro* and *in vivo* antioxidant activity, which could be used as dietary supplements.

## Declare

Each of the authors declares no conflict of interest.
